# The efficacy of ball blankets on insomnia in depression in outpatient clinics: study protocol for a randomized crossover multicentre trial

**DOI:** 10.1186/s13063-020-04638-y

**Published:** 2020-08-17

**Authors:** Sanne Toft Kristiansen, Poul Videbech, Merete Bender Bjerrum, Erik Roj Larsen

**Affiliations:** 1 Research Unit of Nursing and Healthcare, Department of Public Health, Aarhus University, Bartholins Allé 2, Aarhus C and Protac A/S, Niels Bohrs Vej 31D, 8660 Skanderborg, Denmark; 2 Center for Neuropsychiatric Depression Research, Mental Health Centre Glostrup, Glostrup, Denmark; 3 Research Unit of Nursing and Healthcare, Department of Public Health, Aarhus University, Bartholins Allé 2, Aarhus C, Denmark; 4 Mental Health Department Odense – University Clinic, Mental Health Service, Odense, Region of Southern Denmark Denmark; 5 Department of Regional Health Research, University of Southern Denmark, Odense, Denmark

**Keywords:** Insomnia, Depressive disorder, Outpatients, Weighted blankets, Actigraphy, Crossover studies

## Abstract

**Background:**

Depression affects approx. 4% of the global population and is often accompanied by insomnia. Medications used to treat insomnia can have side effects such as development of tolerance and addiction. The Protac Ball Blanket™ (PBB) is a non-pharmacological supplement to sedatives and hypnotics, but evidence for the efficacy of PBB is needed before the treatment is implemented. The objective of this trial is to test the efficacy of PBB on insomnia caused by depression in a randomized controlled design.

**Methods:**

This study is a multicentre, randomized crossover trial with planned inclusion of 45 patients. The randomization procedure is permuted-block randomization with varying block sizes. Patients are allocated into either a sequence “AB” or “BA” each lasting 4 weeks (28 nights). Patients randomized to the “AB” sequence receive treatment A (Protac Ball Blanket™) in the first 2 weeks and switch to treatment B (treatment as usual) in the second period, whereas patients who are randomized to the BA sequence receive treatment B in the first period and treatment A in the second period. The participants will serve as their own control in this design. The primary outcome is changes in total sleep time. Secondary outcome measures are changes in sleep onset latency, number of awakenings, wake after sleep onset, and use of sedatives and hypnotics. Furthermore, quality of sleep, insomnia severity status, and self-reported symptoms of depression, anxiety, interpersonal sensitivity, and neurasthenia will be measured. A paired, two-sided *t* test to compare the means of the differences in the outcomes will be performed.

**Discussion:**

This clinical trial will assess the effect of PBB on depression-related insomnia. The outcomes are of high interest as the PBB is a potential non-pharmacological supplement to medical treatment of patients with insomnia due to depression.

**Trial registration:**

ClinicalTrials.gov Identifier: NCT03730974. Registered on 5 November 2018.

## Background

Depression affects approximately 4% of the global population and is associated with significant morbidity and mortality. Evidence-based treatment of the condition is therefore an important clinical and public health issue [1, 2]. Unfortunately, some symptoms of depression are difficult to manage such as for instance insomnia. Insomnia occurs in the majority of patients with depression [2] and is characterized as problems with initiating sleep, several wake ups, early morning awakening, reduced total sleep time, and daytime dysfunction [3–5], all causing poor sleep quality. The relationship between insomnia and depression is complex. Whether insomnia is part of depression or a separate entity is still debated [6–8]. Insomnia in this trial is addressed as a diagnostic symptom of depression [9]. Possible reasons for insomnia in depression are anxiety, impaired stress regulation, substance abuse, poor sleep hygiene, or ruminations [4, 10]. In both inpatient and outpatient psychiatry, medicine is often used to treat insomnia, but pharmacological treatment has side effects and, importantly, in case of benzodiazepines, there is a risk of drug tolerance and addiction [11] as well as somnolence during day-time. Increased attention on investigating the known and unknown benefits of non-pharmacological treatment options for insomnia due to depression is therefore highly relevant. Non-pharmacological treatments include cognitive behavioural therapy, which has proven effects on insomnia [12–14]. Cognitive behavioural therapy for insomnia (CBT-I) typically includes sleep restriction, stimulus control, cognitive therapy, sleep hygiene, and relaxation training [12–14]. Unfortunately, sleep restriction with formal cognitive restructuring to target hyperarousal, dysfunctional behaviours, and maladaptive beliefs, thoughts, and attitudes is limited to patients without severe cognitive problems who are mentally able to participate in therapy sessions [14, 15]. Participation may therefore be problematic for a number of patients with depression, due to decreased energy and difficulties concentrating and remembering, and in some cases psychotic symptoms. Therefore, additional non-pharmacological treatment options are needed.

The Protac Ball Blanket™ is an option that can be used in all patients including patients with severe cognitive deficits due to depression. It has been shown to decrease sleep disturbances in children with ADHD [16]. Further, it has been used in adult psychiatric inpatient settings in Denmark since the early 1990s for patients suffering from depression, anxiety, agitation, manic episodes, and psychosis. From a clinical perspective, the blanket has been found to enhance the patient’s awareness of the body and its physical delimitation which relieves restlessness, stress, and anxiety and provides a sense of security and calm in mind and body, which for some resolves in improved sleep quality. The theory underlying the reasons for using the PBB for calming purposes is based on theories of Sensory Integration [16–18]. It is hypothesized that the deep pressure stimulation and the sensory inputs from the weight of the blanket are providing sensory input to the Proprioceptive System located in our muscles and joints. This provides us with a sense of body awareness and the movement of the loose plastic balls in the blanket which provide sensory input to the Tactile System reduces the body’s physiological level of arousal and stress, which might improve sleep. Clinical experiences support the use of PBB in depression, but to our knowledge, there are no studies on the exact effects of the blanket and the appropriateness of using PBB in insomnia among adults with depression. Two published dissertations on sleeping problems due to dementia or sense of touch and sense of movement issues in children indicate calming effects of using ball blankets [18, 19]. Overlap in symptoms between these conditions and depression, i.e., anxiety and sleep disturbances, suggest an effect in depression. Currently, ball blankets are used as a non-evidence-based treatment option among adults with depression. However, lack of evidence limits the use in the adult population or may expose some to a useless treatment. Further, if ball blankets are beneficial for a proportion of patients with depression, the lack of data on adult patients hampers further development and improvement of the PBB for the benefit of patients.

An important goal in the treatment of depression is to increase the total sleep time, minimize the sleep onset latency, and improve quality of sleep, and at the same time minimize the use of sedatives [1, 8, 20]. We hypothesize that PBB will extend sleep durations, minimize the sleep onset latency, reduce the number of awakenings, reduce wake after sleep onset, reduce the need for sedatives, improve the quality of sleep, and reduce the self-reported symptoms in patients with insomnia due to depression.

## Methods/design

### Objectives

The objective is to investigate the efficacy of PBB on insomnia caused by depression in a randomized controlled design.

### Hypotheses

The use of PBB on patients with insomnia due to depression will

- Primary goal
  1. Extend the total night-time sleep and
- Secondary goals
  2. Reduce sleep onset latency
  3. Reduce the number of awakenings
  4. Reduce wake after sleep onset
  5. Reduce the need for sedatives
  6. Improve the quality of sleep
  7. Reduce the self-reported symptoms of patients with depression

### Trial design and study setting

This multicentre trial has a randomized 2-sequence, 2-period, 2-treatment crossover design. The rationale for choosing a crossover design is as follows:

1. Traditionally, the recruitment of depressed patients for research trials is heavily challenged due to the nature of depression, and by choosing a crossover design, we can achieve the same precision as a parallel group trial with a lower sample size [21].
2. We further expect easier recruitment and fewer drop-outs as all patients get to try the PBB intervention compared to a parallel group design.

We are not including a washout period in the trial design for one reason:

1. The clinical nature and mechanism of the PBB is unlikely to have a residual effect that persists into the subsequent period, as PBBs only cause effect when patients are exposed to the product.

The data collection period lasts 4 weeks (28 nights in total) with planned inclusion of 45 patients. The study protocol adheres to the SPIRIT (Standard Protocol Items: Recommendations for Interventional Trials) reporting guidelines [22].

The trial takes place in two outpatient settings, The Mood Disorders Clinic, Aarhus University Hospital Psychiatry, Central Denmark Region, and in the Mental Health Department Odense – University Clinic, Mental Health Service, Region of Southern Denmark. Both hospitals are public university hospitals. In Denmark, public hospitals containing outpatient clinics are free of charge and treat all patients in need of urgent treatment of depression.

### Eligibility criteria

Patients will be recruited through their primary contact in the clinics. The project manager (PhD student and psychiatric nurse (STK)) and three research assistants (two psychiatric nurses and a medical student) will perform the interventions.

#### Inclusion criteria

1. Participants: Patients with first depressive episode or with recurrent depressive disorders according to ICD-10 (F32–33) or F32–33 in combination with anxiety disorders F40–41.2 (males and females, aged ≥ 18 years) who receive outpatient treatment in The Mood Disorders Clinic, Aarhus University Hospital Psychiatry, Denmark, and in the Mental Health Department Odense – University Clinic, Mental Health Service, Region of Southern Denmark.
2. Participants must
  Experience poor sleep and have a Global Pittsburgh Sleep Quality Index Score ≥ 5 [3, 5, 14].
  Report one or more of the following:
    - Sleep onset latency ≥ 31 min, occurring ≥ 3 nights a week, for ≥ 14 days, [14, 23]
    - Wake time after sleep onset of ≥ 31 min occurring ≥ 3 nights a week for ≥ 14 days, [14, 23]
    - Early morning awakenings ≥ 3 nights a week for ≥ 14 days [14, 23].

In this trial, early morning awakening is defined as the final morning awakening with a wake-up time ≥ 1 h prior to desired wake up time [14, 24, 25].

#### Exclusion criteria

- Patients that, according to ICD-10 criteria, have been depressed > 2 years
- Patients suffering from hypersomnia (ICD-10: F51.13)
- Patients with harmful use of or dependence on psychoactive drugs (ICD-10: F10–19)
- Patients with diseases directly influencing sleep quality (such as severe chronic pain issues, sleep apnoea)
- Patients who report breathing issues during the eligibility PSQI interview
- Patients with Circadian Rhythm Sleep-Wake Disorders (ICD-10; G47.20–47.26)
- Participation in other research interventions during the intervention period.

### Intervention

The intervention is the 7-kg Protac Ball Blanket™ Flexible cotton 200 cm filled with loose quit plastic balls. The blanket is characterized by the movement of the plastic balls, which provides a changing sensory stimulation. For more information, see www.protac.dk.

Treatment as usual in this trial refers to the regular duvet each patient usually uses when sleeping at home. Information (e.g. size, weight, material) on each of the patient’s duvets will be registered before inclusion for the purpose of describing the average duvets used as control blanket in this trial.

#### Actigraphy

Actigraphy will be used as an instrument to objectively evaluate sleep in this trial.

The devise used is a Motionlogger Micro Watch (Version 1.99.5.1.) from Ambulatory Monitoring Inc. New York, USA. It is a portable device that is the size of a large wristwatch. The actigraph is an accelerometer that detects the intensity and the amount of movements as a function of time [26]. Movements are monitored continuously and stored within the device. Subsequent analysis of frequency and patterns of movement by means of validated algorithms permits detection of sleep-wake patterns. For this trial, movements will be sampled in 1-min epochs. The data activity collection modes are Zero Crossing (ZC) and Proportional Integrating Measure (PIM) [26, 27]. All movements that are scored above a preset threshold using the algorithm Cole-Kripke scoring algorithm are scored as “awake” and those that are below this threshold are scored as “sleep”. The algorithm correctly distinguished sleep from wakefulness 88% of the time when compared to polysomnography [27].

All patients will be asked to wear the actigraph on the wrist of their non-dominant hand for the consecutive 4 weeks (28 nights in total) in their own home environments. As the device is waterproof, patients are instructed to wear the actigraph 24 h a day, including when taking showers. Patients are instructed to press the event-marker as they go to bed, wake up, and when they get out of bed at the start of the next day. These starts and stop intervals will be registered in sleep diaries as patients will be able to visually see if they forgot the registration.

STK and an experienced sleep scientist will perform the scoring and analysis of actigraphic records. The degree of agreement between raters will be calculated and reported using the Intraclass Correlation Coefficient. Data will be analysed using the MotionLogger Analysis Software program Action-W (AW2) (Version 2.7.2).

The pertinent sleep variables chosen for this trial are total sleep time after sleep onset (in minutes) (TST), sleep onset latency (SOL), number of awakenings, and wake after sleep onset (WASO) (for definitions of variables, see the “Outcomes” section).

#### The practicalities of the trial

**Fig. 1:**
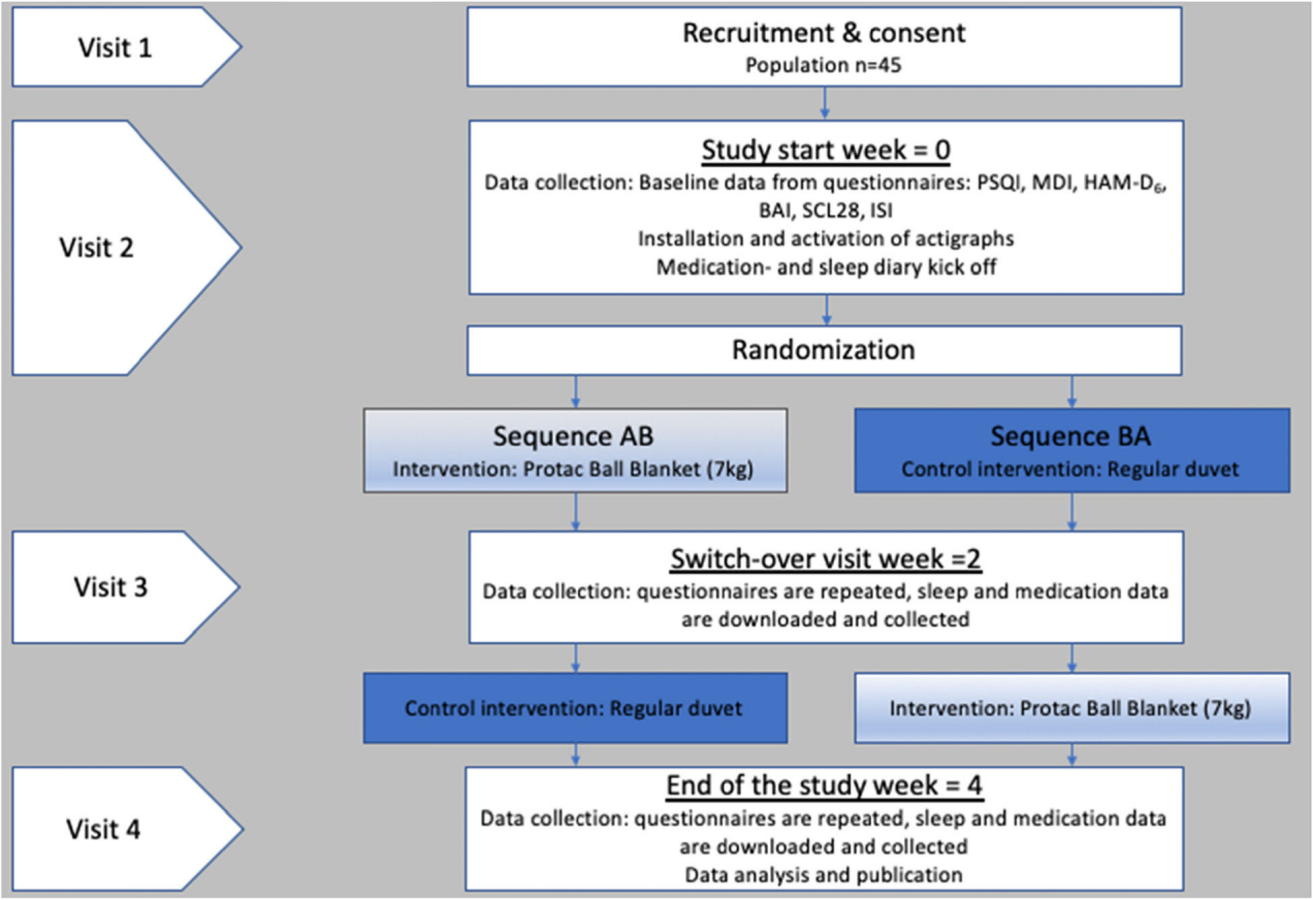
Participant timeline

**Fig. 2:**
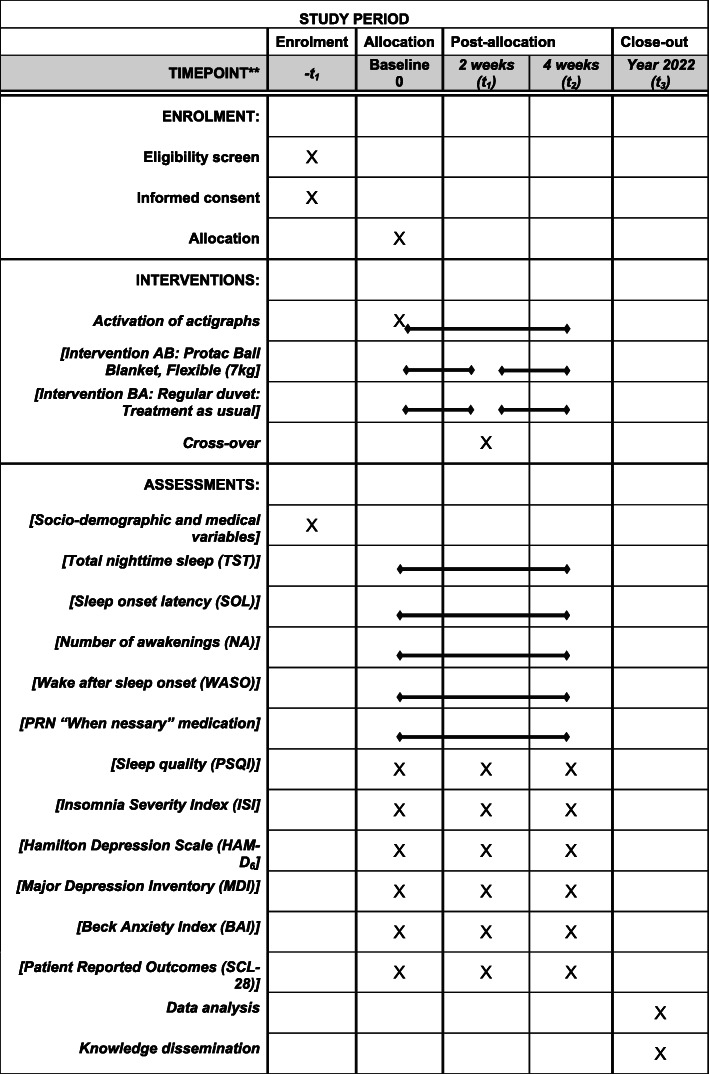
Study schedule for enrolment, intervention, and assessments

Figure 1 outlines the participant timeline, and Fig. 2 shows the standard protocol items recommended by SPIRIT: Schedule for enrolment, intervention, and assessments. Additional file 1 presents The Standard Protocol Items: Recommendations for Interventional Trials (SPIRIT) checklist.

The participant timeline (Fig. 1) shows patients will meet with data collectors four times in total. Initially for the purpose of recruitment and written consent (visit 1), the second visit (Visit 2) consists of the installation and activation of actigraphs. Baseline data will be gathered using six validated questionnaires (see the “Data collection methods” section for further details). Patients will be introduced to the sleep and medication diary (Table 2) presented as a supplementary material. Data from sleep diaries will be used to aid the process of scoring sleep-wake stages, when analyzing patients’ actigraphic sleep records. Subsequently, patients are randomized into either (1) sequence “AB” or (2) sequence “BA” each lasting 4 weeks. Patients randomized to the AB sequence receive treatment A (Protac Ball Blanket™, Flexible, 7 kg) in the first 2 weeks (14 nights) and switch to treatment B (treatment as usual = regular duvet) in the second period, whereas patients that are randomized to the BA sequence receive treatment B in the first period and treatment A in the second period. This means that participants will serve as their own controls.

In both sequences, patients receive general sleep advice prior to participation [4]. The rationale behind this is to avoid behavioural irregularities between periods in order to make comparison of data possible. Patients are recommended to avoid major variations in their sleep-wake patterns from day to day during the trial. But also to avoid major variations in their sleep environment and nicotine, caffeine, alcohol and food consumption directly before bed. Patients are also advised to limit daytime naps to a maximum of 30 min.

The third visit (visit 3) at day 15 consists of a switch-over visit to either receive the intervention or treatment as usual. All six questionnaires will be repeated. Data concerning sleep and medication will be downloaded from actigraphy and collected from diaries for the first 2-week period (14 days and nights). The procedures will be repeated at the end of the study (week four = day 29) at visit 4.

The investigators allow a + 7-day non-compliance with the scheduled visit dates (2 weeks apart) in case patients experience relapse or become ill, which can hinder a physical meeting. The allocated treatment will be discontinued in all cases of patient requests, hospitalization, and alcohol or drug abuse. Patients may withdraw from the trial for any reason. The investigators may withdraw the patient from the study if the patient is unwilling or unable to wear the actigraph, as the analysis of the primary outcome data depends on these measures.

In case patients experience intolerable adverse events and other unintended effects of trial interventions or trial conduct, the reasons will be recorded in the patient’s Case Report Form (CRF) and securely stored in REDCap. All serious adverse events will be reported to the Danish Scientific Ethics Committee, in the final article, and also to Protac A/S.

The overall protocol adherence is secured by STK and the three research assistants. Individual training of research assistants is completed before study initiation. In order to secure full data completion, we have registered all questions from questionnaires as “required” in the REDCap (REDCap 8.5.22 2019 Vanderbilt University) system. Hereby, the system will immediately remind us of any missing data when patients are present. REDCap (Research Electronic Data Capture) is a secure browser-based, metadata-driven EDC software solution and workflow methodology for designing clinical and research databases. All data from this trial will be securely stored in REDCap.

### Outcomes

#### Primary outcome measure

1. Changes in total night-time sleep (TST) after sleep onset measured by actigraphy [time frame: 4 weeks = 28 days]. The investigators will detect the change in total night-time sleep in minutes (i.e. actual sleep time, excluding sleep latency and wakes after sleep onset) by comparing the means of the difference in total sleep time for each participant between the two periods (A and B). The night-time sleep frame is defined as sleep occurring during a 12-h night interval (21:00 to 08:59 h). The investigators designate the first sleep episode after 2100 h as the first nocturnal sleep episode; if the individual is already asleep at that time, the bedtime will be moved earlier to the first epoch of wake prior to sleep onset [26].

#### Secondary outcome measures

1. Changes in sleep onset latency (SOL) will be measured by actigraphy [time frame: 28 days]. The investigators will measure patients’ sleep onset latency in minutes (time between registered bedtime in sleep diaries and first sleep onset measured by actigraphy) and compare the means of the difference for each participant between the two periods (A and B).
2. Changes in number of awakenings measured by actigraphy [time frame: 4 weeks = 28 days]. The investigators will measure the number of awakenings between first sleep onset and the last wake up time registry and compare the means of the difference between periods (A and B) for each participant.
3. Changes in wake after sleep onset (WASO) will be measured by actigraphy [time frame: 4 weeks = 28 days]. The investigators will measure patients’ total time awake between initial sleep onset and the final morning awakening (WASO) in minutes and compare the means of the difference between periods for each participant.
4. Daily use of sedatives and hypnotics will be measured by medication registration use in sleep diaries [time frame: 4 weeks = 28 days]. The investigators will measure the change in total use of per need sedatives and hypnotics in mg by comparing the means of the difference for each participant between periods.
5. Quality of sleep measured by questionnaire using the Pittsburgh Sleep Quality Index (PSQI) [time frame: data will be collected at baseline (visit 2), after 2 weeks (visit 3), and after 4 weeks (visit 4)]. The investigators will measure and report the change in quality of sleep between period A and B. For each participant, the difference between the PSQI measured at the first and the last visit (2 weeks apart) of the exposure period will be subtracted from the difference between the PSQI measured at the first and the last visit (2 weeks apart) of the control period. The total Pittsburgh Sleep Quality Index Score between 0 and 21, with “0” indicating no difficulty and “21” indicating severe difficulties, is reported. Because there will be less than 30 days between assessment at visit 3 and visit 4 and the standard PSQI is a retrospective 30-day instrument, we will use a modified version of the PSQI in which the patients will only be asked about their subjective sleep quality during the past 14 days.
6. Symptoms of depression will be measured by the self-reported Hamilton Depression Rating Scale (HAM-D_6_). Data will be collected at baseline, after 2 weeks, and after 4 weeks. The investigators will measure the change in self-reported symptoms of depression between periods. For each patient, the difference between the scores measured at the first and last visit (2 weeks apart) of the exposure sequence will be subtracted from the difference between the scores measured at the first and last visit (2 weeks apart) of the control period. The total sum scores between 0 and 50 will be reported, where higher values represent worse outcome.
7. Insomnia Severity Status will be measured by questionnaire using the Insomnia Severity Index (ISI). Data will be collected at baseline, after 2 weeks, and after 4 weeks. The investigators will measure the change in patients’ insomnia severity status between periods. For each patient, the difference between the scores measured at the first and last visit (2 weeks apart) of the exposure period will be subtracted from the difference between the scores measured at the first and last visit (2 weeks apart) of the control period. The total scores between 0 and 28 will be reported, where higher values represent worse outcomes.
8. Patients self-reported symptoms of depression measured by questionnaire using the Major Depression Inventory (MDI) [time frame: data will be collected at baseline (visit 2), after 2 weeks (visit 3), and after 4 weeks (visit 4)]. The investigators will measure the change in self-reported symptoms of depression between periods. For each patient, the difference between the scores measured at the first and last visit (2 weeks apart) of the exposure period will be subtracted from the difference between the scores measured at the first and last visit (2 weeks apart) of the control period. The total score between 0 and 50 is reported, where higher values represent worse outcomes.
9. Symptoms of anxiety will be measured by questionnaire using the Beck Anxiety Index (BAI). Data will be collected at baseline (visit 2), after 2 weeks (visit 3), and after 4 weeks (visit 4).The investigators will measure the change in self-reported symptoms of anxiety between periods. For each patient, the difference between the scores measured at the first and last visit (2 weeks apart) of the exposure period will be subtracted from the difference between the scores measured at the first and last visit (2 weeks apart) of the control period. The total score between 0 and 63 for each visit is reported, where higher values represent worse outcomes.
10. Patient-reported outcomes concerning interpersonal sensitivity, neurasthenia, anxiety, and depression will be measured by questionnaire using The Self-Reported Symptom State Scale (SCL-28) [time frame: data will be collected at baseline (visit 2), after 2 weeks (visit 3), and after 4 weeks (visit 4)]. The investigators will measure the change in self-reported symptoms between period A and B or B and A. For each patient, the difference between the scores measured at the first and last visit (2 weeks apart) of the exposure period will be subtracted from the difference between the scores measured at the first and last visit (2 weeks apart) of the control period. Sub scales are combined to compute a total score. The range for each sub scale is: Interpersonal Sensitivity 0–32, Neurasthenia 0–28, Anxiety 0–32, and Depression 0–24. Both total score and sub scale scores will be reported. The total score ranges between 0 and 112, where higher value represents worse outcomes. Because of an overlap in one question between the sub scales anxiety and depression question, number 31 is only considered in the total sum scores.

### Sample size

The power calculation is based on data from a pilot study (*N* = 8) as we have not identified any studies investigating the effects of ball blankets on TST in depressed patients.

The main outcome will be changes in total night-time sleep with and without PBB estimated by the means of the differences between the periods for each participant. The null hypothesis is that there is no change in sleep duration. In the pilot study, the mean sleep duration appeared normally distributed and the mean sleep duration without a ball blanket was 420 min. The standard deviation of the difference between the sleep duration with and without a ball blanket was 42 min. Based on the potential clinical gains, we aim to be able to detect a change in total sleep duration of 20 min. Assuming that the differences in sleep duration with and without a ball blanket is normally distributed, we performed power calculations for a paired *t* test, and with an alfa = 0.05 and power = 0.80 the minimum required sample size is *N* = 37. To take into account dropout rates, we aim to include 45 patients.

### Recruitment

Patients will be recruited through dedicated teams of nurses, doctors, and psychologists at each hospital. STK or research assistants will participate in weekly treatment conferences in order to help the nurses identify participants according to the eligibility criteria. When potential participants are identified, the primary contact nurse will perform the first contact. She will briefly inform them about the project and hand out the leaflet “Ball blankets as a treatment option for sleep disturbances”. In cases where patients show interest, the primary contact nurse will ask for oral permission for STK or one of the research assistants to make contact with them by phone. The contact nurse will also ask for permission to pass on the following information to STK or the research assistants from their medical journal before signing the written consent for participation: their phone number, ICD-10 diagnosis, actual medication prescriptions, and symptoms of sleep disturbances. Shortly after, STK or the research assistants will contact the patient and go over the inclusion criteria on the phone in order to verify the patients’ ability to participate. Further, patients will receive oral information on the aim, methods, benefits, and consequences of participation by phone and will be asked for permission to receive written patient participation material by e-mail. This enables the patients to read the material in quiet surroundings in cooperation with a relative. The material is concisely and precisely formulated in order to be easily understood by patients with potential cognitive deficits. Three to 5 days later, STK or one of the research assistants will make contact with the patients in order to confirm or disconfirm their interest in arranging a meeting. At a meeting, oral information on the project will be repeated and questions will be answered for the purpose of gaining written consent. Before signing the consent, each patient will be given a 1-week period for deliberation. The consent will give STK or the research assistants the right to gather the relevant information necessary for the analyses, i.e. medication use and diagnosis. The information will be given by STK or the research assistants in a quiet and undisturbed meeting room at the hospital in the presence of a relative. No further information from patients’ medical journal will be gathered after the signing of the written consent unless patients inform us of changes in diagnosis or medication prescriptions. The written consent will be uploaded and stored in REDCap.

If the inclusion rate is unexpectedly low, additional outpatient clinics from other university hospitals will be included.

### Allocation

The randomization procedure is permuted-block randomization with varying block sizes of 4, 6, and 8 generated by an independent service provider using REDCap for randomization, meaning detailed information on blocking is unavailable to those who enrol participants and assign interventions. STK and the research assistants have access to the randomization module in REDCap as data will be entered and stored in REDCap. STK and the research assistants will be informed of allocation sequences by pressing the randomization button in REDCap. The allocation sequence is not concealed from the outcome assessors (STK and the research assistants).

### Blinding

The administration of blankets will be performed in a randomized order but blinding of the patients and operators is not possible. The data analyses will be performed blinded to the sequence (“AB” or “BA”, described above).

### Data collection methods

The following data will be collected for the purpose of the research and recruitment:

1. From hospital records: Name, phone number, personal identification number from the Danish Civil Registration System (CPR number), diagnosis, medication prescriptions.
2. From actigraphy (Motionlogger Micro Watch from Ambulatory Monitoring Inc. NY): Total sleep time, sleep onset latency, number of awakenings, wake after sleep onset.
3. From diaries: The use of sleep and anxiety medications, bedtime and time of getting out of bed, number of awakenings and duration, wake after sleep onset and daytime naps including lengths registered in minutes.
4. From six validated questionnaires: The Global PSQI Score using The Pittsburgh Sleep Quality Index (PSQI), the insomnia severity score using the Insomnia Severity Index (ISI), depressive symptoms using the Major Depression Inventory (MDI) and 6-item Hamilton Rating Scale for Depression (HAM-D_6_), patients’ self-reported symptoms using the Self-reported Symptom State Scales (SCL-28) and anxiety symptoms using the Beck Anxiety Index (BAI) [3, 25, 28–30].

### Data management

All data will be securely stored in REDCap. Using the function “identifier” in REDCap enables the full trial dataset to be anonymized when exported to the Stata IC15 and MotionLogger Analysis Software programs. Sleep data from actigraphy will not include any personal identifiers. Sleep data will be stored under the allocated ID number from REDCap.

The trial will be conducted in adherence to the Declaration of Helsinki - Ethical principles for medical research involving human subjects.

### Statistical methods

Paired, two-sided analyses will be performed. If the differences in the measures between the exposure and control periods for each patient are normally distributed, as we expect, we will use a Student’s *t* test to test the null hypotheses of no differences. For the non-normal distributions, non-parametric tests, e.g. Wilcoxon signed-rank tests or Fisher’s exact tests will be used.

All data with or without protocol violation will be included in the analysis with complete outcome data for all randomized participants. Because the analyses are based on the differences between period A and B or B and A for each patient, we will perform complete case analysis (i.e. participants with missing actigraphic data from one of the periods will be removed from the analysis). An intention to treat analysis will be performed, i.e. patients that in one or both of the periods use an “incorrect”, non-study blanket will be kept in the analyses in their randomized sequence group. We will not account for missing data by imputation.

The level of statistical significance will be set to 5%.

The randomization will determine whether the participants will begin or end with a PBB. The baseline characteristics of these two groups will be presented in a table. Baseline information on sleep will be based on The Pittsburgh Sleep Quality Index (PSQI) and the Insomnia Severity Index (ISI) and not collected via actigraphy.

There will be no wash-out period in this trial because the PBB are expected to have no residual effect persisting in the subsequent periods. This assumption is based on the biological mechanisms of the ball blanket and on clinical experience, but there are no studies to corroborate this expectation. This aspect of the design, however, is expected to lead to bias toward the null, i.e. that the study will not overestimate a potential effect of PBB. The assumption of no carry-over effect in the data will be ruled out in a preliminary test. The sum of the TST values will be calculated for the time period when using PBB and when using patients’ own duvets for each participant. This will be compared across the sequence groups by means of an independent *t* test. If this test yields a statistically significant result, the usual test for differences between the effects of treatment A or B will not be applied [31].

Data will be analysed using Stata IC15 (Stata Corp, College Station, TX, USA).

### Data monitoring

No data monitoring committee (DMC) will be established for this trial. Further, no interim analysis will be performed due to the short duration of the trial and a minimal intervention risk.

## Discussion

This trial has some limitations. First, we use the PSQI four times during the trial. For the purpose of screening for eligibility and again at visits 2, 3, and 4. However, PSQI is a retrospective 30-day instrument and there will be less than 30 days between assessments at visits 3 and 4 (14 days to be precise) [3]. However, there will be no overlap in the recall periods between visits, as patients are asked about their sleep quality during the past 14 days at these particular two visits using a modified version of the PSQI. This is not expected to cause any bias to the results and interpretations of these as a duration of 2 to 3 weeks is often recommended clinically to differentiate transient from persistent sleep-wake disorders [3]. Most importantly, the PSQI with a 1-month recall period will be used as suggested when screening for eligibility and again at visit 2, which will enable us to separate the transient sleep-wake problems from the persistent sleep-wake problems when recruiting patients for this trial [3].

Second, remission in the underlying depression condition may occur during the trial. In order to minimize the chance of remission and to ensure a constant intensity during both periods (AB and BA), we have chosen a short intervention period. The condition is less likely to develop radically compared to when there are long treatment periods.

Third, patients in outpatient clinics are often in need of acute treatment. Therefore, period effects may occur due to newly introduced medication or medication adjustments during the trial period. Nevertheless, patients will not be excluded from the study due to these conditions as recruitment will then become almost impossible. Besides, such conditions will be equally distributed between sequence groups A-B and B-A due to the randomization. In order to interpret the results critically, we register medication prescriptions at baseline and again in sleep diaries if any changes are made during the trial period.

In summary, this is a randomized crossover multicentre trial testing the efficacy of Protac Ball Blankets™ on insomnia in depression treated in an outpatient setting. An easy identification of the included patients by clearly defined inclusion criteria, together with the multicentre design, supports the broad clinical relevance of the study results. Because both treatments are evaluated for the same individual, the treatment effect can be estimated based on an average of within-individual differences. Hereby, the crossover design removes between-patient variation and patients can indicate clear preferences for PBB vs regular duvets. The outcome of this trial is of high interest as the PBB may be a potential non-pharmacological supplement to medical treatment of patients with insomnia due to depression.

## Trial status

Recruitment of participants began on November 21, 2019, and will be completed in May 2021. The manuscript reports protocol version 5, October 9, 2019.

The results will be submitted for publication in an international peer-reviewed open access journal in Spring 2022. Authors will have to meet the principles of the Vancouver Declaration. The results will be presented at both national and international conferences, fairs, and hospitals. Furthermore, the results will be presented at national and international business collaboration meetings by means of Protac A/S contacts. An e-mail will inform the patients about test results if requested.

## Supplementary information

**Additional file 1.** SPIRIT 2013 Checklist: Recommended items to address in a clinical trial protocol and related documents.

**Additional file 2.** Sleep and medication diary.

## Data Availability

Not applicable.

## Abbreviations

AMI: Ambulatory Monitoring Inc.
BAI: Beck Anxiety Index
HAM-D6: 6-Item Hamilton Rating Scale for Depression
ISI: Insomnia Severity Index
MDI: Major Depression Inventory
PBB: Protac Ball Blanket™
PIM: Proportional Integrating Measure
PSQI: The Pittsburgh Sleep Quality Index
SCL-28: Self-reported Symptom State Scales
SOL: Sleep onset latency
TST: Total sleep time
WASO: Wake after sleep onset
ZC: Zero Crossing
