# Supplementary figures and images for: The efficacy of ball blankets on insomnia in depression in outpatient clinics: study protocol for a randomized crossover multicentre trial
Source: Trials. 2020 Aug 17;21:720. doi: 10.1186/s13063-020-04638-y (PMC7430021; doi:10.1186/s13063-020-04638-y)

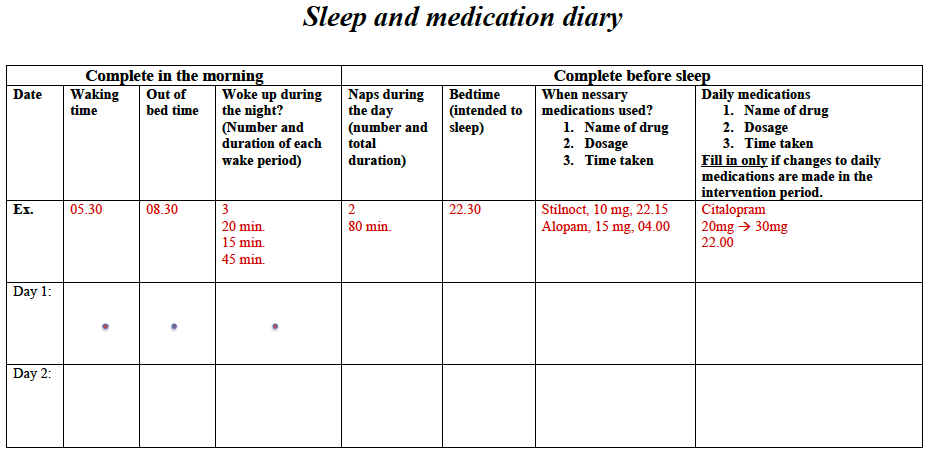

Supplement: Supplementary file 2 — Additional file 2. Sleep and medication diary. [file 13063_2020_4638_MOESM2_ESM.docx]
